# Reduction in ambulatory visits for acute, uncomplicated bronchitis: an unintended but welcome result of the coronavirus disease 2019 (COVID-19) pandemic

**DOI:** 10.1017/ice.2020.1233

**Published:** 2020-09-28

**Authors:** Thomas J. Dilworth, Charles F. Brummitt

**Affiliations:** AdvocateAuroraHealth, Milwaukee, Wisconsin

**Fig. 1.**
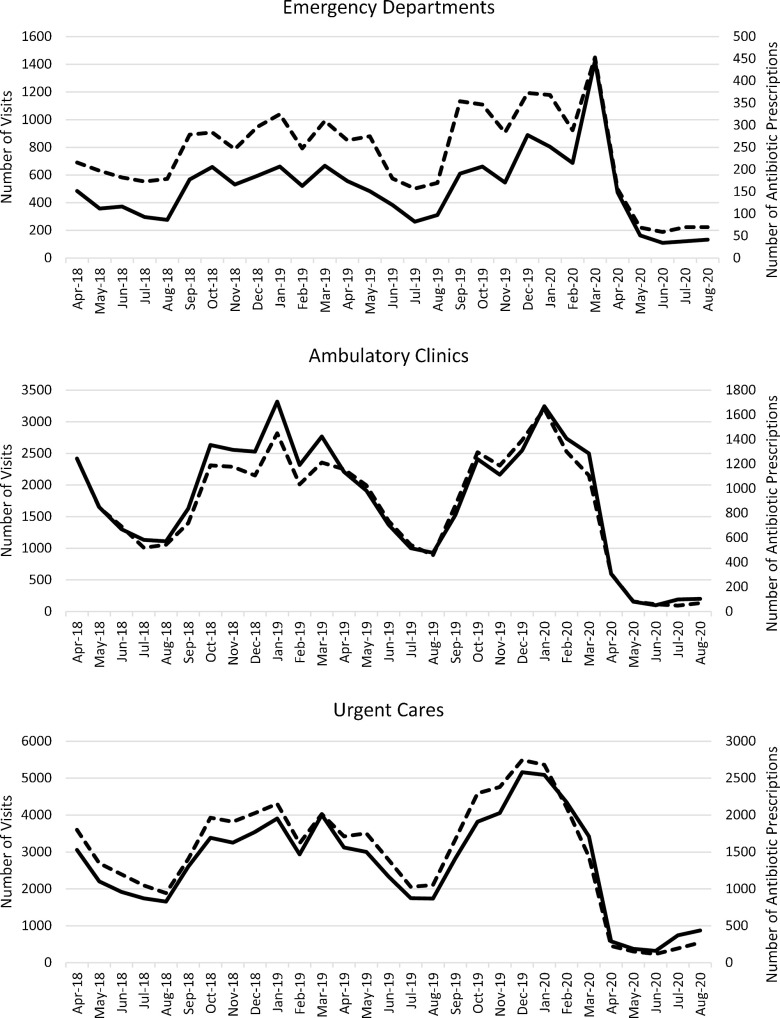
Number of ambulatory patients with a primary diagnosis of bronchitis, April 2017–August 2020. Complete lines, number of visits; dashed lines, number of antibiotic prescriptions.

*To the Editor*—The coronavirus disease 2019 (COVID-19) pandemic has adversely impacted nearly all aspects of life since emerging in late 2019. Notably, surges in COVID-19 cases have led to antibiotic overprescribing in the inpatient setting, hampering ongoing antimicrobial stewardship efforts.^1,2^ However, patients’ reluctance to seek healthcare during the pandemic, particularly for minor ailments, may lead to unexpected outpatient antimicrobial stewardship gains. Jeffery et al^3^ reported an inverse relationship between COVID-19 cases and daily counts of emergency department visits in 5 US states between January and April of 2020. Diagnoses were not reported in this study, and the authors concluded that clinicians should reinforce to patients the importance of seeking emergency department care for serious conditions. We have previously reported the results of a systemwide initiative to reduce antibiotic prescribing for ambulatory adults with acute, uncomplicated bronchitis.^4^ Internally, we continue to track and report these data. Recently, we observed a profound reduction in both the overall number of patients seen and discharged with a primary diagnosis of bronchitis and the number of antibiotic prescriptions written for these encounters (Fig. 1). In addition to patients’ not seeking care due to the pandemic, there are a number of other, possible explanations for our observed decline in visits. Stay-at-home orders and social distancing appear to have reduced the burden of common respiratory viruses in the community, leading to fewer cases of acute, uncomplicated bronchitis,^5,6^ for which the primary etiology is viral.^7^ Patients may increasingly seek care outside of our health system during the pandemic. The pandemic has also shifted patient care to telemedicine. To assess this phenomenon, we captured whether or not a visit was a telemedicine encounter and then analyzed the recent ambulatory clinic data. Of the 394 ambulatory clinic bronchitis visits during July and August, 112 (28.4%) were telemedicine encounters, a higher proportion of total visits than observed in previous months. Thus, a shift toward more telemedicine visits was revealed in our data set.

The COVID-19 pandemic, while challenging for so many other aspects of antimicrobial stewardship, has led to an overall net reduction in ambulatory adults seeking care for bronchitis in our health system and to a dramatic reduction in antibiotic prescribing for that condition. Reducing unnecessary, outpatient antibiotic prescribing has long been a difficult challenge for many antimicrobial stewardship programs. Our challenge will be to educate patients and clinicians to maintain these improvements in outpatient, acute bronchitis management as the pandemic is brought under control and outpatient visits return to prepandemic levels.
